# A novel tubular construct using silk fibroin-chitosan scaffold seeded with bone marrow mesenchymal stem cells for urinary diversion in a preclinical rabbit model

**DOI:** 10.3389/fsurg.2026.1867284

**Published:** 2026-06-23

**Authors:** Qianliang Wang, Qingling Liu

**Affiliations:** 1 Department of Urology, Affiliated Hospital of Guangdong Medical University, Zhanjiang, China; 2 Department of Medical Records, Affiliated Hospital of Guangdong Medical University, Zhanjiang, China

**Keywords:** bone marrow mesenchymal stem cells, chitosan, scaffold materials, silk fibroin, tissue engineering, urinary diversion

## Abstract

**Objective:**

To construct tissue-engineered tubular grafts (TETGs) using bone marrow mesenchymal stem cells (BMSCs) and silk fibroin/chitosan (SF/CS) scaffolds, and to evaluate their feasibility and efficacy for urinary diversion in a rabbit model.

**Methods:**

BMSCs, smooth muscle cells (SMCs), and urothelial cells (UCs) were isolated and characterized *in vitro*. Differentiated BMSCs and SMCs were seeded onto SF/CS scaffolds for an additional week. The resulting TETGs were implanted for urinary diversion in the experimental group (*n* = 24), while acellular SF/CS grafts served as controls (*n* = 24). Histological and immunohistochemical analyses were performed at 1, 2, 4, and 8 weeks post-implantation. Outflow tract patency was assessed via intravenous urography (IVU) at 10 weeks.

**Results:**

BMSCs exhibited high expression of CD44 and CD90 and low expression of CD34 and CD45. BMSCs successfully differentiated into urothelium-like cells, expressing the urothelial-specific proteins uroplakin 1A (UP1A), cytokeratin 7 (CK7), and cytokeratin 13 (CK13). All rabbits in the experimental group survived, showing luminal urothelial growth, with complete epithelialization achieved by week 8. Immunohistochemistry confirmed the presence of mature and functional epithelial cells, as evidenced by positive staining for Anti-cytokeratin AE1/AE3, UP1A, and the tight junction protein ZO-1. IVU confirmed patent outflow tracts without stenosis, urinary fistula, hydronephrosis, or ureteral dilation. In contrast, control grafts collapsed by week 4, resulting in mortality.

**Conclusion:**

BMSCs can serve as a viable cell source for urinary tissue engineering. The construction of a urinary diversion conduit using BMSC-derived urothelium-like cells and SF/CS is feasible in rabbits, demonstrating its potential for clinical application.

## Introduction

Pathological alterations of the urinary bladder can lead to impaired bladder function or necessitate bladder resection, often requiring subsequent bladder reconstruction procedure (1, 2), gastrointestinal segments are the most frequently used materials for such reconstruction in clinical practice. In urinary diversion procedures, one end of these conduits is connected to the ureters to collect urine, while the other end forms a stoma on the abdominal wall for urinary excretion. However, the use of gastrointestinal segments is associated with considerable long-term complications, such as urinary tract infections, stone formation, metabolic disturbances, and an increased risk of malignancy (3–5). Moreover, the harvest of gastrointestinal tissue inflicts injury on an otherwise healthy alimentary tract, exposing patients to additional surgical risks, including anastomotic leakage and stricture (6, 7). Therefore, there is growing interest in developing tissue-engineered conduits cultured *in vivo* as a promising alternative that may eventually replace gastrointestinal segments in future clinical practice.

Tissue engineering applies engineering principles to the development of biological substitutes for damaged tissues, with a core methodology centered on creating functional grafts *in vitro* using cells and scaffolds (8). Among various scaffold materials, silk fibroin/chitosan composites have been extensively studied and shown to support the attachment, growth, and proliferation of diverse cell types (9–11). Nevertheless, the clinical translation of urothelial cell-based grafts faces challenges, particularly regarding the safety of harvesting autologous urothelial cells from patients with bladder diseases, including those with oncological histories. As an alternative cell source, bone marrow-derived mesenchymal stem cells (BMSCs) offer advantages in terms of accessibility and safety. Our previous work has demonstrated the successful use of BMSCs in reconstructing ureteral tissue in a rabbit model (12). Although the direct use of stem cells in tissue engineering has been debated due to their differentiation potential and possible tumorigenic risks (13), studies such as that by Tian et al. have confirmed that BMSCs can be induced to differentiate into urothelium-like cells, suggesting their utility as a viable cell source in urological tissue engineering (14).

Despite advances in tissue engineering, few studies have successfully constructed a functional, cell-seeded tubular graft for urinary diversion that achieves both complete epithelialization and sustained patency in a preclinical model. Most previous efforts have focused on acellular scaffolds or single-cell-type seeding, often resulting in graft collapse, stenosis, or insufficient epithelial coverage (15). To address these gaps, the present study introduces several novel elements: (1) the use of BMSC-derived urothelium-like cells as a safe and accessible cell source, avoiding the oncological risks associated with autologous urothelial cells from bladder cancer patients; (2) a silk fibroin/chitosan composite scaffold with optimized porosity and mechanical properties tailored for urinary conduit construction; and (3) a two-step *in vivo* preconditioning strategy (omental wrapping followed by urinary diversion) to promote neovascularization and epithelial maturation. This combination has not been previously reported for urinary diversion.

## Materials and methods

### Animals

All animal procedures were approved by the Laboratory Animal Ethics Committee of Guangdong Medical University (Approval No: GDY2302199) and conducted in accordance with institutional guidelines. Male New Zealand white rabbits (2.0–2.5 kg) were obtained from the Experimental Animal Center of Guangdong Medical University.The animals were housed individually in standard stainless steel rabbit cages to prevent aggression and allow for individual monitoring. Each cage was equipped with a resting grid.Rabbits were housed under controlled environmental conditions: temperature 22 ± 2 °C, relative humidity 50 ± 10%, and a 12 h/12 h light/dark cycle. They were kept individually in stainless steel cages (60 × 60 × 50 cm) with wood shavings as bedding, which was changed twice weekly. Standard rabbit chow and tap water were provided *ad libitum*.The rabbits had *ad libitum* access to a standard laboratory rabbit pellet diet and autoclaved tap water via automatic drinking nipples.Specifically, for sacrifice, a lethal dose of sodium pentobarbital (120 mg/kg) was administered intravenously following a standardized protocol approved by our Institutional Animal Care and Use Committee. Loss of consciousness was confirmed by the absence of corneal reflex, followed by cardiac arrest.

### Anesthesia methods

All surgical procedures were performed under general anesthesia. Rabbits received an intravenous injection of 3% pentobarbital sodium (30 mg/kg) through the marginal ear vein. The depth of anesthesia was monitored by assessing pedal withdrawal reflex, heart rate, and respiratory rate.

### Post-operative care

After urinary diversion surgery, rabbits were placed in a heated recovery cage (30 °C) and monitored closely until fully conscious. Post-operative pain was managed with buprenorphine (0.02 mg/kg, subcutaneously) administered every 8 h for the first 72 h, and carprofen (2 mg/kg, subcutaneously) once daily for 5 days. All animals received prophylactic antibiotics: enrofloxacin (5 mg/kg, intramuscularly) once daily for 7 days. The urinary catheter placed in the stoma was maintained for 7 days post-surgery; the stoma site was cleaned daily with sterile saline and povidone-iodine solution. Rabbits were housed individually in sterilized cages with soft bedding, which was changed daily during the first post-operative week to prevent infection. Food and water were provided *ad libitum*, and body weight, food intake, urine output, stoma appearance, and general behavior were monitored daily for the entire study period. Any signs of distress, infection, urinary leakage, or obstruction were recorded. All procedures were approved by the Institutional Animal Care and Use Committee and performed in accordance with institutional guidelines for animal welfare.

### Euthanasia methods

At the end of the experiment, animals were deeply anesthetized with an intramuscular injection of ketamine (40 mg/kg) and xylazine (5 mg/kg). Subsequently, euthanasia was performed by intravenous injection of pentobarbital sodium (120 mg/kg) into the marginal ear vein, followed by exsanguination and bilateral thoracotomy to ensure death, in accordance with the AVMA Guidelines for the Euthanasia of Animals.

### Isolation and culture of BMSCs and SMCs

BMSCs were harvested from the rabbit tibia. Briefly, following venipuncture of the marginal ear vein, a Fr14 bone marrow aspiration needle was inserted proximal to the tibia. Approximately 5 mL of bone marrow was aspirated into a heparinized syringe. The marrow was mixed with PBS containing antibodies, centrifuged at 1,500 rpm for 10 min, and the pellet was resuspended and centrifuged again. The resulting cell pellet was resuspended in DMEM supplemented with 10% FBS and plated at a density of ≥5 × 10^5^ cells/mL. Cells were cultured at 37 °C with 5% CO₂, with medium changes every 2–3 days. Upon reaching 80% confluence, cells were passaged. Third-passage BMSCs were characterized by flow cytometry. Briefly, cells were detached using 0.25% trypsin-EDTA, washed twice with PBS, and resuspended in staining buffer (PBS containing 2% FBS and 0.1% sodium azide) at a concentration of 1 × 10^6^ cells/mL. Cells were incubated with the following antibodies for 30 min at 4 °C in the dark: mouse anti-rabbit CD44-FITC (clone: W3/13, 1:100 dilution), mouse anti-rabbit CD90-PE (clone: L13, 1:100 dilution), mouse anti-rabbit CD34-PE (clone: 43A1, 1:50 dilution), and mouse anti-rabbit CD45-FITC (clone: L12, 1:100 dilution). Isotype-matched mouse IgG1-FITC and IgG1-PE (1:100 dilution) were used as negative controls. After incubation, cells were washed twice with staining buffer and fixed with 1% paraformaldehyde. Flow cytometric analysis was performed on a BD FACSCalibur flow cytometer (BD Biosciences, San Jose, CA, USA) equipped with a 488 nm argon laser. The gating strategy was as follows: cell population was first gated on forward scatter (FSC) vs. side scatter (SSC) to exclude debris and doublets, followed by FSC-H vs. FSC-A gating to select single cells. A minimum of 10,000 events were acquired for each sample. Data were analyzed using FlowJo software (version 10.0, Tree Star, Ashland, OR, USA). The percentage of positive cells was determined based on the isotype control threshold (<2%).Rabbit BMSCs were isolated using the whole bone marrow adherence method as previously described (16).

SMCs were isolated from rabbit bladder wall biopsies using a tissue explant method (12). Muscle tissues were explanted into Petri dishes containing DMEM with 10% FBS and cultured at 37 °C with 5% CO₂. After seven days of expansion, cells were digested and passaged. SMCs from passages 3–5 were used for experiments.

### Isolation, culture, and identification of urothelial cells

Bladder tissue was minced, and the mucosal layer was separated and digested with 0.25% trypsin for 30–60 min at room temperature. The digest was centrifuged at 1,500 rpm for 5 min, and the cell pellet was resuspended in keratinocyte serum-free medium (KSFM). Cells were cultured at 37 °C with 5% CO₂ and passaged as needed. For identification, cells were trypsinized, cytospun onto slides, and incubated with primary antibody CK AE1/AE3 (1:200 dilution) overnight at 4 °C, followed by a FITC-conjugated secondary antibody. Staining was visualized using a fluorescent inverted microscope.Rabbit urothelial cells were isolated using a trypsin digestion method as previously described (14).

### Induction and detection of BMSC differentiation (transwell method)

A Transwell co-culture system (Corning) was employed for this study. The system consists of upper and lower chambers separated by a polycarbonate membrane with a pore size of <3 μm, which prevents cell migration while allowing the free passage of cytokines and small molecules. Passage 3 BMSCs and urothelial cells (UCs) in the logarithmic growth phase were harvested. Briefly, cells were detached using 0.25% trypsin containing 0.02% EDTA for 1 min. The digestion was stopped by adding complete DMEM medium. After centrifugation at 1,000 rpm for 5 min, the cell pellets were resuspended in their respective complete culture media to prepare single-cell suspensions. For the co-culture, BMSCs were seeded in the lower chamber at a density of 1 × 10^5^ cells/cm^2^ in DMEM supplemented with 10% fetal bovine serum (FBS). Concurrently, UCs were seeded in the upper chamber at a density of 2 × 10^5^ cells/cm^2^ in keratinocyte serum-free medium (KSFM). A control group, consisting of BMSCs in the lower chamber without any cells in the upper chamber, was established in parallel. Following stratified seeding, the Transwell plates were placed in a humidified incubator at 37 °C with 5% CO₂ for indirect co-culture over a period of two weeks. The medium was replaced every three days. Cells were passaged when they reached approximately 90% confluency.

### Preparation and evaluation of silk fibroin-chitosan composite scaffolds

Silk fibroin solution was prepared as follows. Bombyx mori silk cocoons were cut into small pieces and degummed by boiling twice in 0.5% (w/v) Na₂CO_3_ solution for 30 min each time. The degummed silk fibers were thoroughly rinsed with distilled water and air-dried overnight. The dried fibers were dissolved in 9.3 M LiBr solution at 60 °C for 4 h with gentle stirring. The resulting solution was dialyzed against distilled water using a cellulose membrane (molecular weight cutoff: 3.5 kDa) for 48 h at room temperature, with water changes every 6 h. The dialyzed silk fibroin solution was concentrated to 6% (w/v) using polyethylene glycol (PEG, 20 kDa) and stored at 4 °C for further use.

Chitosan powder (degree of deacetylation ≥95%, molecular weight 50–190 kDa) was dissolved in 2% (v/v) acetic acid at room temperature with continuous stirring at 500 rpm for 6 h. The solution was filtered through Whatman No. 1 filter paper to remove undissolved particles, resulting in a final chitosan concentration of 2.5% (w/v).

The silk fibroin and chitosan solutions were mixed at a volume ratio of 6:4 (SF:CS). This ratio was selected based on preliminary optimization experiments, which demonstrated the best balance of mechanical strength (elastic modulus: 28.10 ± 1.58 MPa) and porosity (93.52% ± 3.68%) for urinary conduit applications. The mixture was stirred gently for 30 min to ensure homogeneity and then degassed under vacuum to remove air bubbles.

The composite mixture was poured into cylindrical molds (diameter 10 mm) and subjected to directional freezing using a copper rod immersed in liquid nitrogen to create aligned pores. The freezing protocol consisted of holding at −20 °C for 2 h, followed by −80 °C for 12 h. The frozen samples were then lyophilized using a lab-scale freeze dryer at −55 °C under vacuum pressure below 0.1 mbar for 48 h.

To enhance stability in aqueous environments, the lyophilized scaffolds were treated with 95% ethanol for 30 min, followed by neutralization with 0.5% sodium hydroxide for 15 min, and then washed extensively with distilled water. No chemical crosslinkers were used to avoid potential cytotoxicity.

For sterilization, scaffolds were immersed in 75% ethanol for 2 h, washed three times with sterile PBS, and exposed to UV irradiation overnight in a biosafety cabinet prior to cell seeding.

### Seeding cells onto the CS/SF scaffolds

After 14 days of induction, differentiated cells were adjusted to a concentration of 4 × 10^6^ cells/mL and seeded dropwise onto one side of the scaffold. SMCs (passage 3–5) were seeded onto the opposite side. The cell-scaffold constructs were cultured in DMEM for one week, with daily medium changes. Cellular proliferation was assessed via H&E staining, SEM, and Live/Dead viability assay. The seeded scaffold was then wrapped around a 10F catheter and tailored to a 6 cm length to form the TETGs.

### Transferring TETGs into the omentum of rabbits

Under general anesthesia, twenty-four TETGs were implanted into the rabbit omentum and allowed to integrate for two weeks prior to urinary diversion surgery. Six acellular CS/SF tubular scaffolds were similarly implanted as a control group. After two weeks, grafts were harvested for histological analysis (H&E and immunohistochemical staining) to assess epithelial regeneration and neovascularization.

### Implanting TETGs into the rabbits for urinary diversion

Rabbits were anesthetized with 3% sodium pentobarbital. A midline abdominal incision was made, the bladder was exposed and completely resected, and the urethra was sutured closed. The bilateral ureters were anastomosed to the TETGs in a simple continuous pattern. The TETGs was tunneled through the peritoneum, with its distal end forming an abdominal wall stoma. A catheter was placed in the stoma for urinary drainage for one week. In the control group, the same procedure was performed using acellular CS/SF scaffolds (*n* = 6).

### Histologic analysis and intravenous urography assessment

Six rabbits from the experimental group were euthanized at 1, 2, 4, and 8 weeks post-transplantation, and the TETGs were harvested for analysis. Control group rabbits were also analyzed. Tissue sections (5 μm thick) were prepared from formalin-fixed, paraffin-embedded samples for H&E and immunohistochemical staining. Epithelial layers were assessed using antibodies against cytokeratin AE1/AE3, zonula occludens-1 (ZO-1), and uroplakin IIIa. Smooth muscle and neovascularization were identified using antibodies against *α*-SMA and CD31, respectively. Intravenous urography (IVU) was performed 10 weeks postoperatively to evaluate the kidneys, ureters, and TETGs.

### Statistical analyses

Statistical analyses were performed using SPSS Statistics version 19.0. Continuous data with a normal distribution are presented as mean ± standard deviation (x ± s). Comparisons between two groups were conducted using the independent samples t-test. For comparisons among multiple groups, one-way analysis of variance (ANOVA) or the Kruskal–Wallis test was applied, as appropriate. *post-hoc* pairwise comparisons were performed using the Bonferroni correction. A *p*-value of less than 0.05 was considered statistically significant.

## Results

### Morphological characterization and identification of cells

**Figure 1:**
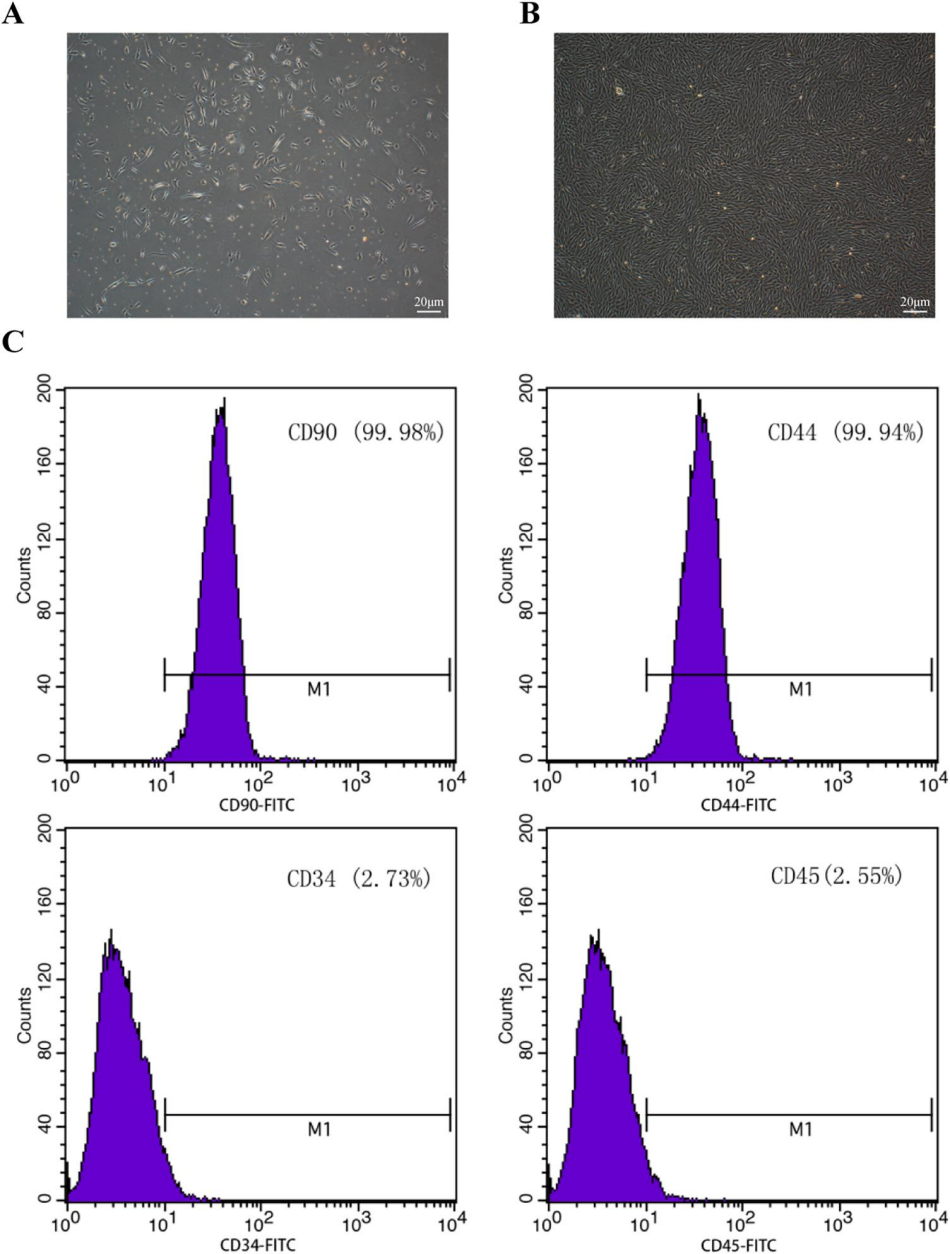
Morphological characterization and identification of BMSCs. **(A)** Primary culture after three days (x100). **(B)** Cells after 10 days of incubation, showing rapid proliferation and a spindle fibroblastic appearance (x100). **(C)** Flow cytometry analysis demonstrating high-level expression of CD90 (99.98%) and CD44 (99.94%), and low-level expression of CD34 (2.73%) and CD45 (2.55%).

Primary BMSCs appeared round initially and adopted a spindle-shaped morphology within 3 days (Figure 1A). By day 10, cells reached 90% confluence, displaying a typical fibroblastic appearance (Figure 1B). Flow cytometry of passage 3 (P3) cells confirmed a BMSC phenotype, with high expression of CD44 (99.94%) and CD90 (99.98%), and low expression of CD34 (2.73%) and CD45 (2.55%) (Figure 1C).

**Figure 2:**
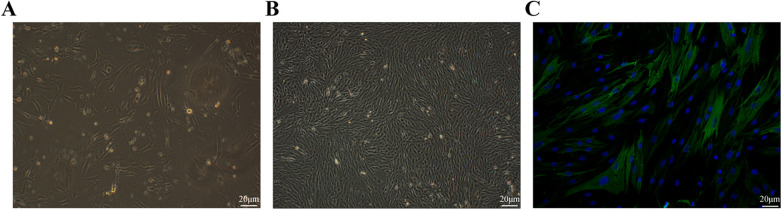
Morphological characterization and immunofluorescence staining of SMCs. **(A)** Primary culture after three days, showing about 30% confluence (x100). **(B)** Cells after seven days, reaching 80%–90% confluence with a classic spindle-shaped morphology (x100). **(C)** Immunofluorescence staining for *α*-SMA (green) and DAPI (blue) (x200).

SMCs reached approximately 30% confluence after 3 days of primary culture (Figure 2A) and 80%–90% confluence with a spindle-shaped morphology by day 7 (Figure 2B). Immunofluorescence confirmed high expression of *α*-SMA (Figure 2C).

**Figure 3:**
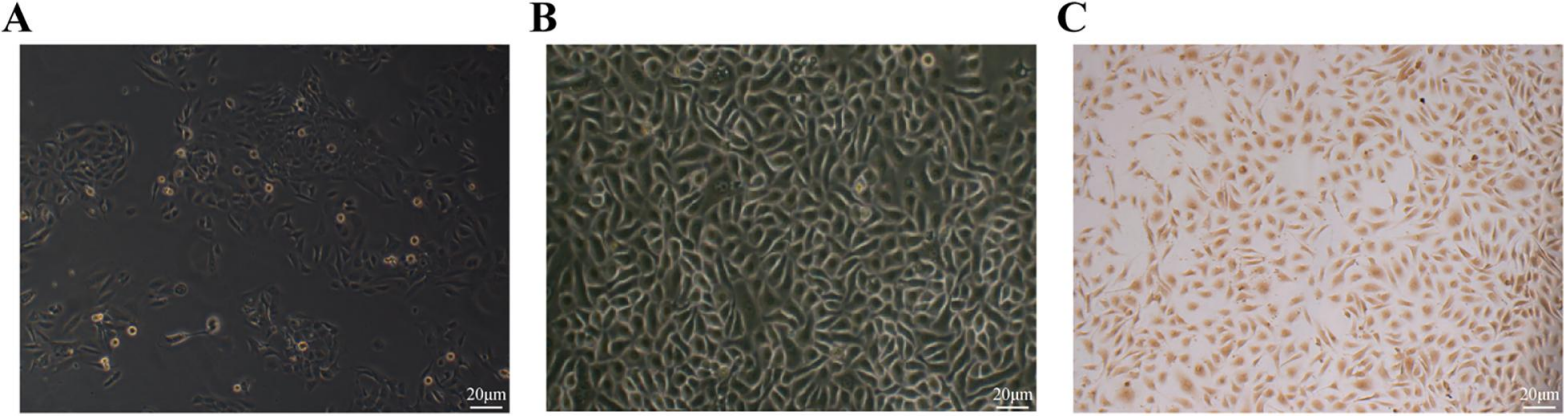
Urothelial culture and identification. **(A)** Primary culture of urothelial cells at 3 days (x100). **(B)** Confluent culture of urothelial cells at 12 days, showing cobblestone morphology (x100). **(C)** Immunofluorescence identification of bladder epithelial cells with anti-CK AE1/AE3 (×200).

Urothelial cells adhered and began forming small colonies by day 3 (Figure 3A). By day 12, cells reached confluence, exhibiting a characteristic cobblestone morphology (Figure 3B). Immunofluorescence with anti-CK AE1/AE3 antibodies confirmed their epithelial identity (Figure 3C).

### Characteristics and phenotypic identification of cells after induced differentiation

**Figure 4:**
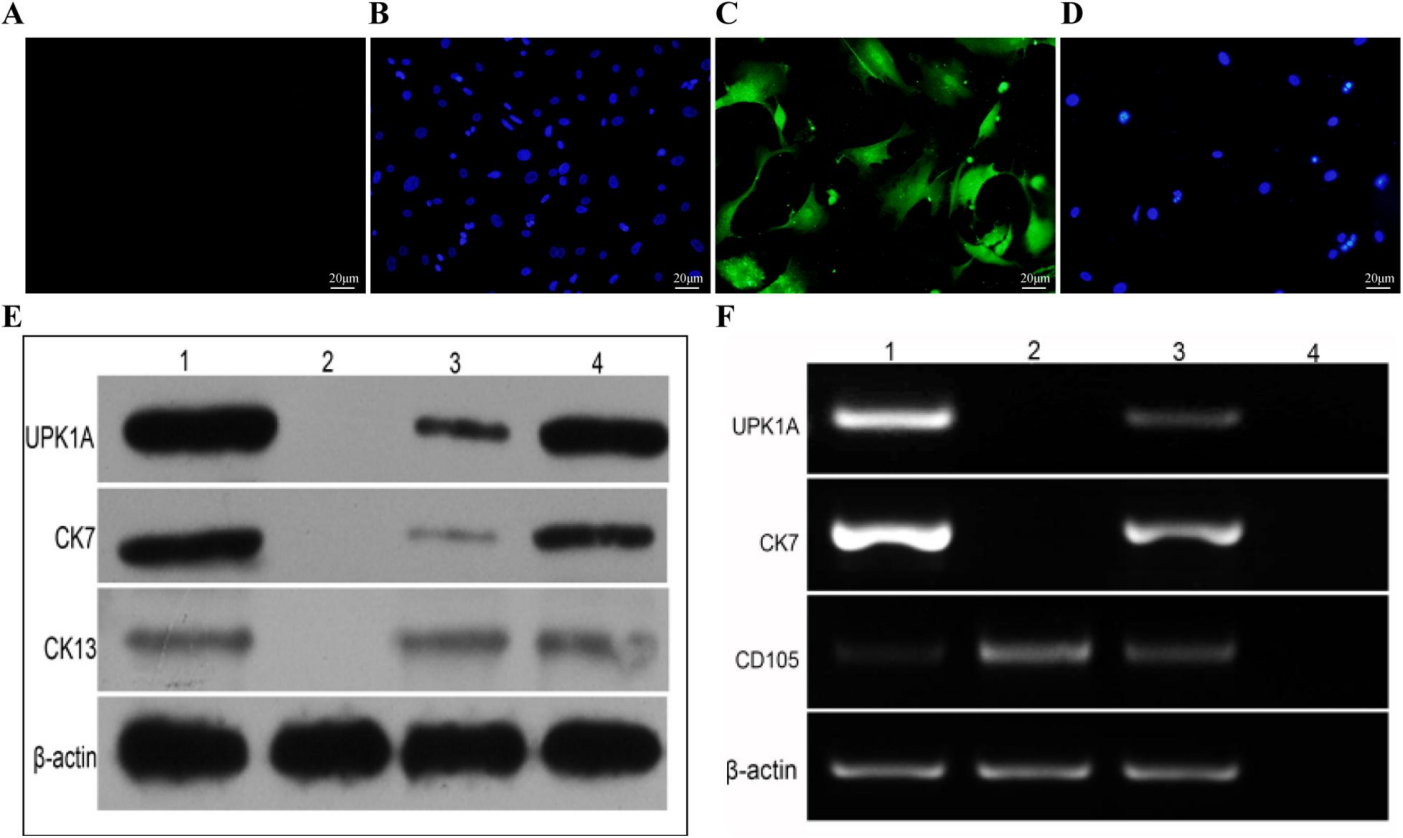
Characterization and phenotype identification of BMSCs after induction. **(A,B)** Third-generation BMSCs do not express UPIa (×200). **(C,D)** Induced differentiation cells express UPIa after indirect co-culture (×200). **(E)** Western Blot analysis of UPkIa 、CK-7 and CK-13 protein expression. Lane 1: urothelial cells; Lane 2: induced BMSCs; Lane 3: non-induced BMSCs. **(F)** RT-PCR analysis of UPKIa and CK-7 gene expression. Lane 1: urothelial cells; Lane 2: induced BMSCs; Lane 3: non-induced BMSCs.

Immunofluorescence revealed that uninduced BMSCs did not express uroplakin Ia (UPIa) (Figures 4A,B), whereas induced cells showed positive UPIa expression (Figures 4C,D). Western blot analysis confirmed upregulated protein expression of UPIa, CK-7 and CK-13 in induced cells compared to non-induced BMSCs (Figure 4E). RT-PCR further demonstrated increased mRNA expression of UPIa and CK-7 post-induction (Figure 4F), confirming successful differentiation of BMSCs into a urothelial phenotype.

### Observation of the silk fibroin-chitosan composite scaffold

**Figure 5:**
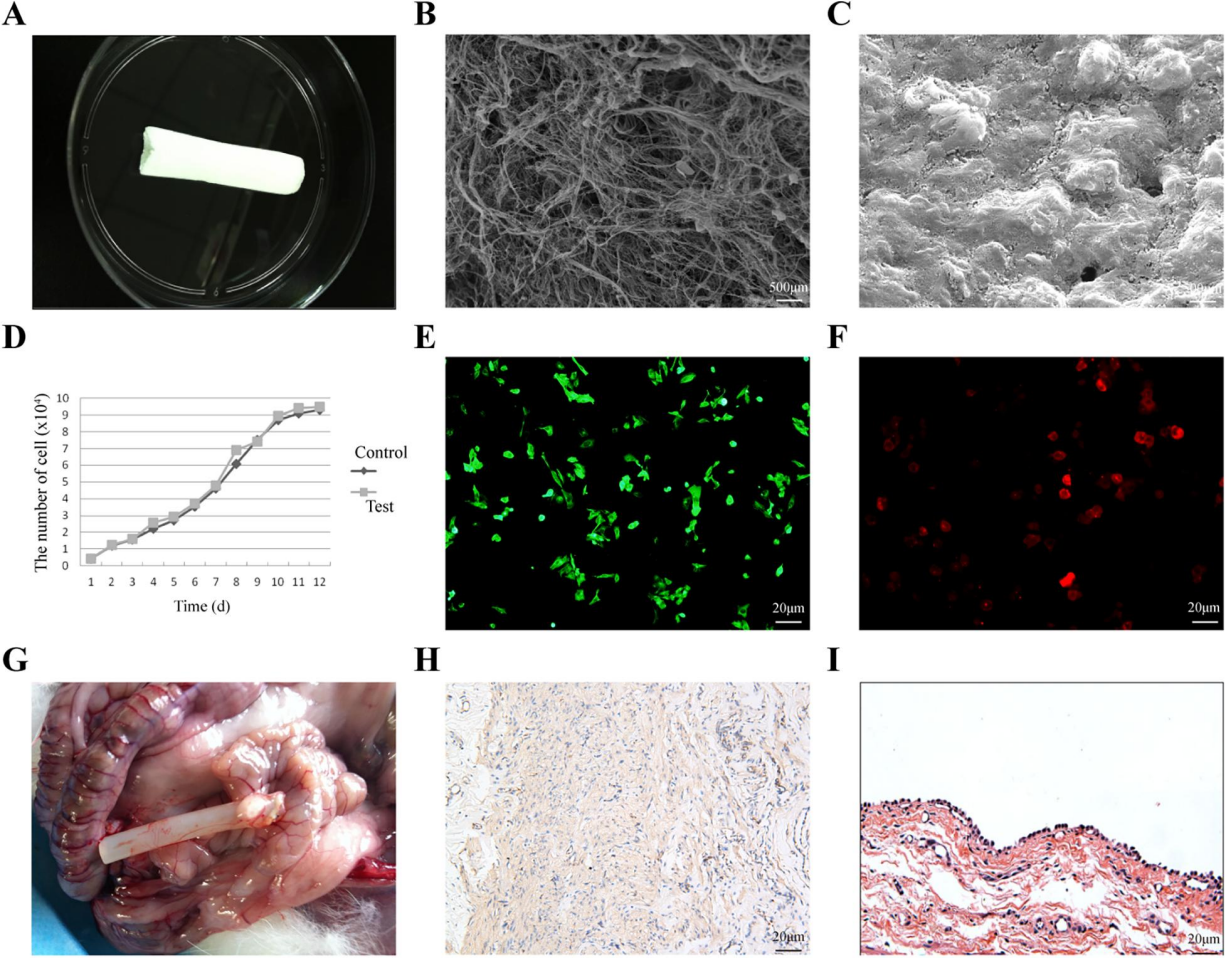
Characteristics of CS/SF scaffold and induced cell biocompatibility. **(A)** Lyophilized scaffold. **(B)** SEM image of the scaffold microstructure. **(C)** SEM image of cells on the scaffold after 3 days of co-culture. **(D)** cell proliferation on CS/SF Scaffold. **(E)** Live viability assay. **(F)** Dead assay **(G)**Macroscopic view of the scaffold 2 weeks after omental wrapping, showing neovascularization. **(H)** CD31 immunohistochemistry confirming angiogenesis. **(I)** H&E staining showing a thin layer of urothelial cells covering the graft lumen.

The freeze-dried SF/CS scaffolds were white (Figure 5A). SEM revealed a uniform, fishnet-like porous structure with pore diameters ranging from 130 to 175 μm(mean pore size of 155.78 μm) (Figure 5B). The average porosity was 93.52% ± 3.68%,the significant water absorption capacity 143.15% ± 5.97%. The scaffold also displayed an elastic modulus of 28.10 ± 1.58 MPa and a compressive strength of 0.65 ± 0.02 MPa, indicating an ability to resist certain pressures and allow for surgical handling.

### Post-induction cell biocompatibility with CS/SF scaffold

SEM on day 3 of culture showed cells adhering and spreading on the scaffold, extending pseudopods and forming intercellular bridges (Figure 5C). Cell growth curve on scaffolds indicated that cell proliferation in the experimental group accelerated after day 7, surpassing the control group by day 9 (Figure 5D). Live/Dead staining confirmed high cell viability (>90%) on the scaffold (Figures 5E,F).

### The histological and immunohistochemical results of the tissue-engineered tubular grafts after omentum wrapping

Macroscopic observation two weeks post-omental wrapping revealed new blood vessels on the graft surface (Figure 5G). Immunohistochemistry for CD31 confirmed significant neovascularization (Figure 5H). H&E staining indicated a thin layer of urothelial cells covering the luminal surface (Figure 5I).

### Histological and intravenous urography evaluation

**Figure 6:**
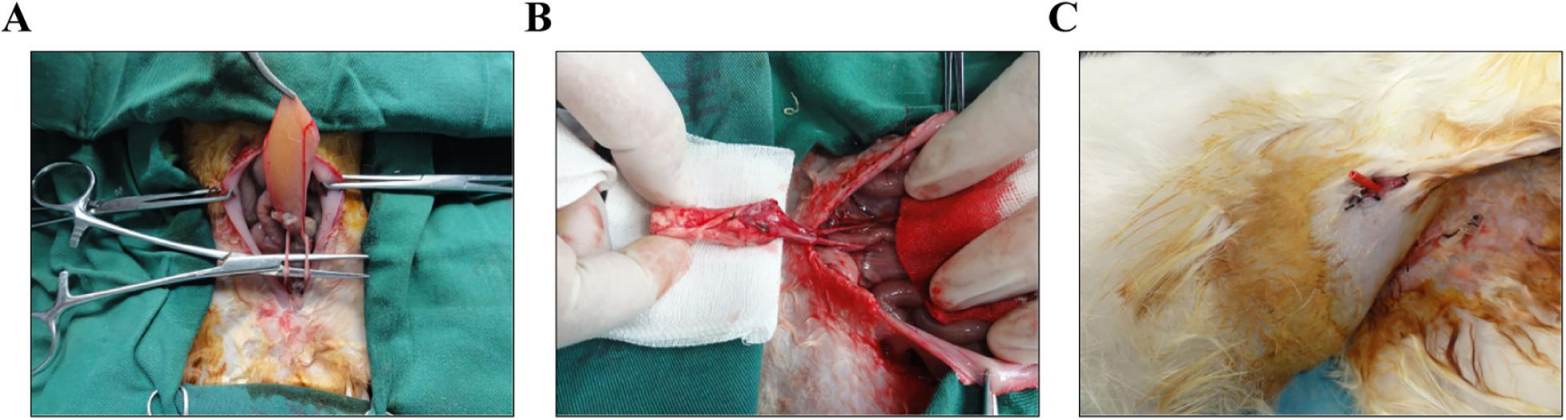
Urinary diversion procedure in rabbits. **(A)** Dissociation of the bilateral ureters. **(B)** Anastomosis of the bilateral ureters to the TETSs. **(C)** Gross observation after urinary diversion surgery.

**Figure 7:**
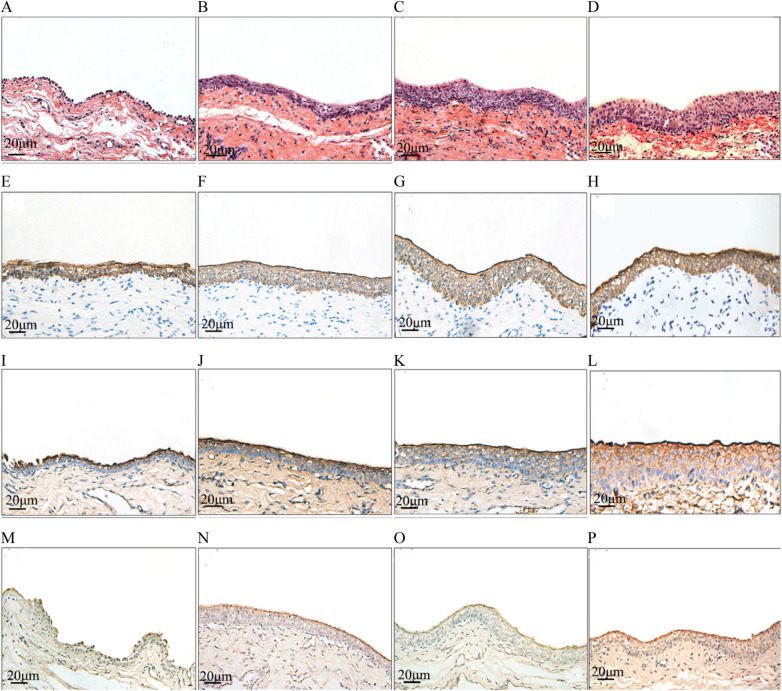
Histologic characteristics of TETSs in the experimental group at 1, 2, 4, and 8 weeks post-diversion. **(A–D)** H&E staining showing regeneration of the epithelial layer (x400). **(E–H)** Immunohistochemical staining for AE1/AE3 (x400). **(I–L)** Immunohistochemical staining for uroplakin IIIa (x400). **(M–P)** Immunohistochemical staining for ZO-1 (x400).A, E, I, M: 1 week post-operation; B, F, J, N: 2 weeks post-operation; C, G, K, O: 4 weeks post-operation; D, H, L, P: 8 weeks post-operation.

**Figure 8:**
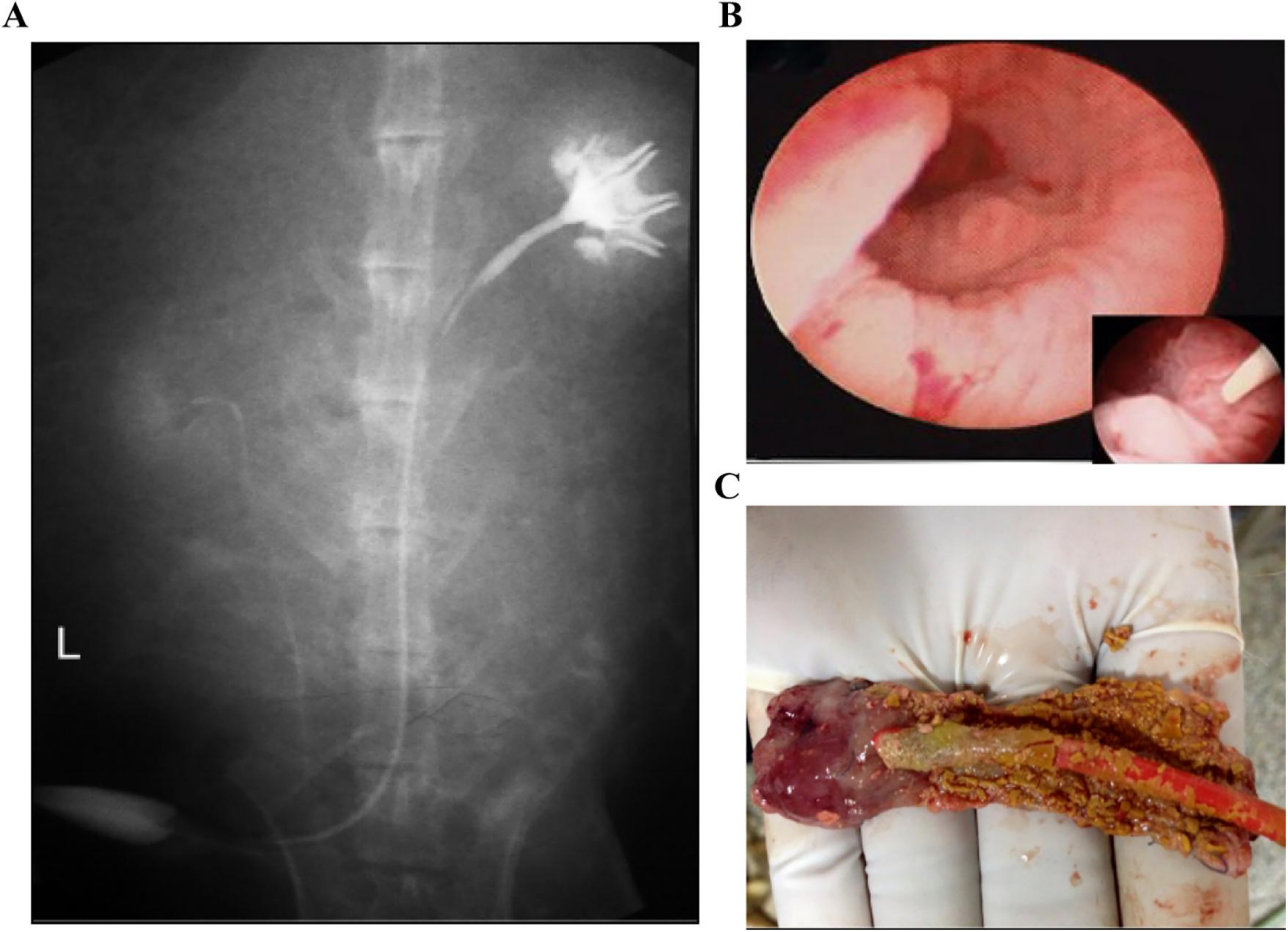
Intravenous urography and related observations. **(A)** IVU at 10 weeks postoperatively showing a patent urinary tract without leakage, stricture, or obstruction. **(B)** Cystoscopic view showing a smooth luminal surface. **(C)** Control group, showing stone formation at 2 weeks post-diversion.

All 24 rabbits in the experimental group survived the urinary diversion procedure, with no observed ureteral dilation, hydronephrosis, urinary leakage, or significant graft contraction (Figures 6A–C). In contrast, all six control rabbits died within 4 weeks. H&E staining of the experimental TETGs showed a thin, loose layer of cells at one week (Figure 7A), a thickened and tightly arranged epithelial layer at two weeks (Figure 7B), and complete luminal coverage by week 4 (Figure 7C), which was maintained at week 8 (Figure 7D). Immunohistochemistry for AE1/AE3 and UPIIIa showed discontinuous positive expression at week 1, becoming continuous and stronger by weeks 2, 4, and 8 (Figures 7E–L). ZO-1 expression followed a similar increasing and strengthening pattern over time (Figures 7M–P). IVU at 10 weeks demonstrated normal renal secretion, no ureteral dilation, and patent, non-stenotic TETGs without contrast extravasation (Figures 8A,B).autopsy revealed collapsed outflow tracts with crystalline deposits on the luminal surface (Figure 8C).

## Discussion

Urinary diversion, achieved via an ileal conduit, pouch, or neobladder, is a standard procedure following radical cystectomy. Tissue-engineered urinary conduits present a promising alternative, offering the potential to reduce operative time and circumvent serious complications associated with bowel resection. In this study, we developed a tubular graft using urothelium-like cells derived from bone marrow-derived mesenchymal stem cells (BMSCs) and a silk fibroin/chitosan (SF/CS) composite scaffold, mimicking the bowel segments conventionally used in urostomy. The performance of this construct was evaluated in a rabbit model. All implanted conduits remained patent and demonstrated watertight integrity, both within the lumen and at the ureter–conduit anastomotic sites.

The urothelium serves as a critical barrier, preventing bacterial invasion, urinary leakage, and protecting underlying tissues. However, the safety of utilizing autologous urothelial cells from bladder cancer patients for tissue engineering remains uncertain due to potential oncological risks (17). Therefore, we explored BMSCs as a safe and viable alternative source of seeding cells. Prior studies have indicated that BMSCs can differentiate into urothelium-like cells when exposed to conditioned medium or through co-culture with urothelial cells, showing upregulation of urothelium-specific genes and proteins (18). Our results corroborate these findings, confirming the successful differentiation of BMSCs into urothelium-like cells *in vitro*.

The composition of the conditional culture medium proved crucial for directing this differentiation. While BMSCs are typically maintained in low-glucose Dulbecco's Modified Eagle Medium (L-DMEM) supplemented with 10% fetal bovine serum (FBS), urothelial cells are generally cultured in serum-free or low-serum media such as Keratinocyte Serum-Free Medium (KSFM). We observed that urothelial cell proliferation was significantly inhibited in media containing 3% FBS or higher. Conversely, BMSCs failed to differentiate in serum-free KSFM. After systematic optimization, a conditional medium consisting of a 4:1 mixture of L-DMEM to urothelial cell-conditioned medium, with 2% FBS, was established as optimal. Following the transition to this conditional medium, a reduction in cell growth rate was noted, accompanied by a morphological shift from a refractive, fibroblastic appearance to a flatter, more epithelial-like phenotype. Over two weeks, the cells became shorter and broader, with a loss of pseudopodia.

Immunophenotypic analysis revealed that the expression of stem cell markers CD90 and CD44 was detectable in induced cells but significantly downregulated after conditional medium treatment. Concurrently, the differentiated cells began to express urothelium-specific markers, including uroplakin 1A (UP1A), cytokeratin 7, and cytokeratin 13, confirming their epithelial transformation. These results indicate that the induced BMSCs adopted a urothelium-like phenotype *in vitro*. While these cells may not represent fully mature urothelial cells, they were capable of forming a continuous, functional barrier layer within the conduit lumen, analogous to native urothelium.

The formation of tight junctions, essential for barrier function (19), was assessed via immunohistochemistry for Zonula Occludens-1 (ZO-1). Positive staining for ZO-1 demonstrated the establishment of tight intercellular connections in the engineered epithelium after implantation, confirming the presence of a cohesive, intact epithelial layer rather than a collection of isolated cells.

The selection of an appropriate scaffold is paramount in tissue engineering. Ideal scaffold materials should be hydrophilic to facilitate nutrient absorption and cell seeding, possess adequate mechanical strength to withstand physiological pressures, exhibit excellent biocompatibility to support cell proliferation and tissue integration, and undergo controlled biodegradation without interfering with new tissue formation. Furthermore, they should be cost-effective, readily available, and easy to process. The SF/CS composite used in this study meets these criteria. Both silk fibroin and chitosan are natural polymers known for their excellent cytocompatibility and histocompatibility, as well as their biodegradable properties. Previous studies have demonstrated that SF/CS scaffolds support the attachment, proliferation, and migration of various cell types, including endothelial cells and stem cells (20, 21). Our scaffold characterization revealed a mean pore size of 155.78 μm, providing a sufficient three-dimensional structure for cell infiltration and growth. The high porosity (93.52% ± 3.68%) ensures adequate oxygen and nutrient diffusion, while the significant water absorption capacity (143.15% ± 5.97%) aids in nutrient delivery. The scaffold also displayed an elastic modulus of 28.10 ± 1.58 MPa and a compressive strength of 0.65 ± 0.02 MPa, indicating an ability to resist certain pressures and allow for surgical handling. Its elasticity and 3D architecture help prevent contact inhibition observed in 2D cultures. However, it is noteworthy that the rigidity of the scaffold was relatively low.

Although our short-term observations confirmed that the BMSC-seeded SF/CS conduits could function effectively for urinary diversion, we observed fibroblast deposition beneath the epithelium layer. This suggests a potential risk for stricture formation in longer-term applications. This finding is consistent with a report by Geutjes et al. (22), who observed hydronephrosis and hydroureter in a porcine model using a collagen-polymer conduit. We hypothesize that the lack of an anti-reflux mechanism in these engineered conduits leads to elevated ureteral pressure, resulting in hydroureter and subsequent hydronephrosis. The incorporation of a functional anti-reflux structure is therefore critical for maintaining the physiological pressure gradient from the kidney to the bladder and represents a key challenge for future research before clinical translation can be considered. Furthermore, to better mimic the native ileum segment, the introduction of additional cell types, particularly smooth muscle cells (SMCs), may be necessary. Several studies (23, 24) emphasize the importance of SMCs in replicating the structural and functional properties of the urinary tract, including contractile activity. Notably, BMSCs have also shown the potential to differentiate into smooth muscle cells and promote SMC proliferation (25, 26), making them a promising single source for multiple cell lineages in bladder tissue engineering. Research in this direction is currently underway in our laboratory.

This technique holds the potential to offer patients requiring urinary diversion a novel alternative: the creation of autologous, tissue-engineered conduits using their own BMSCs and an SF/CS scaffold. This approach could ultimately replace the use of intestinal segments, thereby eliminating associated complications and preserving healthy bowel tissue.

This study has several limitations. First, Given the small caliber of rabbit ureters, and to minimize the potential confounding effects of surgical anastomotic technique, the surgical procedure was modified by preserving the bladder trigone and anastomosing the tubular graft directly to it. This approach likely contributed to the absence of complications such as ureteral-conduit anastomotic stricture or urinary leakage during the follow-up period. Second,The follow-up period (8–10 weeks) is relatively short for a model in which stricture formation, fibrosis, or scaffold degradation may occur. To enhance the clinical relevance of our findings, future work will involve large animal models where the surgical procedure will closely replicate the clinical ileal neobladder technique, thereby establishing a more representative model for evaluating long-term safety and efficacy. We will aslo evaluating these very issues—degradation kinetics, long-term patency, and fibrosis—over an extended period (e.g., 6–12 months) will be a central objective of our planned large-animal (porcine) studies, which are designed to better model the clinical timescale and anatomy.

## Conclusions

In this study, we have produced a tissue-engineered conduit using urothelium-like cells derived from BMSCs and SF/CS, in a rabbit model. We observed that BMSCs could be transformed into urothelium-like cells that can then be proliferated *in vitro*. The urothelium-like cells have the ability to form an epithelium layer on SF/CS similar to the internal surface of the natural urinary tract. Over time, this epithelium layer became thicker and the junctions between cells became stronger. Although certain aspects of the present studies' design needs improvement and the tissue-engineered conduit is still far from being a clinical application, it represents an attractive potential choice for future urinary diversion surgery.

## Data Availability

The raw data supporting the conclusions of this article will be made available by the authors, without undue reservation.
